# An innovative submucosal filler for esophageal endoscopic submucosal dissection: A porcine model study (with video)

**DOI:** 10.1371/journal.pone.0331618

**Published:** 2025-09-11

**Authors:** LiLin Lin, SuYu Chen, Rui Huang, JianHua Chen, Juan Lin, Hong Shi

**Affiliations:** Department of Endoscopy Center, Clinical Oncology School of Fujian Medical University, Fujian Cancer Hospital, Fujian Branch of Fudan University Shanghai Cancer Center, Fuzhou, Fujian, China; Shanghai Jiao Tong University Medical School Affiliated Ruijin Hospital, CHINA

## Abstract

**Background:**

Esophageal endoscopic submucosal dissection (ESD) a more challenging procedure. Numerous studies have explored submucosal injection materials from diverse perspectives. However, there is no consensus yet, and further exploration is still required. This study aimed to evaluate the efficacy of a novel submucosal filler, acetylcysteine, which is commonly used in respiratory medicine, when applied for the first time in ESD procedure of the porcine esophagus.

**Methods:**

A self-controlled survival study was carried out on porcine model. In each pig, the novel submucosal filler was injected in one part of the esophagus (Experimental group; Group E), while normal saline was injected in another part (Control group; Group C) during the ESD procedure. Additionally, one pig received a submucosal injection of normal saline as a negative control to observe baseline daily movements and pathology following ESD. One month post- ESD, endoscopic and pathological evaluations were conducted to assess wound healing and other vital organs, allowing for a comprehensive assessment of the long – term impact and efficacy of the filler in this novel application context.

**Results:**

The dissection time per unit area in Group E was notably shorter than in Group C (3.79 ± 1.38 minutes vs. 5.79 ± 1.22 minutes; *P* = 0.004). Group E also required a smaller volume of submucosal filler per unit area than Group C (3.48 ± 1.07 ml vs. 7.48 ± 3.96 ml; *P* = 0.039). However, There were no significant differences between Group E and Group C regarding the complication rate and thickness of the submucosal layer in the ESD specimens. Pathological evaluation of wound healing and other vital organs demonstrated comparable results in Group E and Group C.

**Conclusion:**

This novel submucosal filler can expedite the ESD process without increasing the incidence of complications.

## Introduction

Endoscopic submucosal dissection (ESD) is a crucial therapeutic approach for early gastrointestinal cancers and precancerous lesions, offering the benefits of minimal invasiveness and having curative potential [1,2].

The main challenges in ESD arise during the submucosal dissection process, particularly in preventing damage to blood vessels and the muscular layer [3,4]. The esophageal wall is thin due to the lack of a physiological serosal layer [5], which increases the risk of complications. Complications associated with ESD include hemorrhage, perforation, coagulation syndrome, and postoperative stenosis [3]. Current research aimed at easing the challenges of ESD focuses on two main aspects: firstly, improving the design of specific devices, such as electrosurgical knives [6] to make them safer and more effective, as an electrosurgical current can burn the muscular layer or transmural of the gastrointestinal tract [6]. Secondly, the development of ESD specific injection products that maintain a long-lasting mucosal cushion to ensure good visualization and access to the submucosal space. Our research direction is to employ blunt mechanical separation to enhance the efficiency of submucosal dissection, thereby reducing the duration of electrosurgery and the associated risk of complications.

The novel submucosal filler developed with acetylcysteine as the main ingredient. Acetylcysteine has not been applied in ESD. In China, acetylcysteine has a long history and is widely used in respiratory medicine as a mucolytics [7], which acts by disrupting disulfide bonds [7]. Hirsch demonstrated that nebulized acetylcysteine, administered at 10–20% concentrations, can significantly reduce sputum consistency [8]. Data from clinical studies and extensive use in clinical practice and as over-the-counter supplementation have demonstrated that oral NAC is generally well-tolerated and safe, even at high doses [7]. These features might contribute to disconnection of submucosal connective tissues by producing a blunt separation effect, leading us to hypothesize that it may be used as an submucosal filler in ESD. In this study, we conduct experiments using a porcine model to assess the efficacy and safety of this novel submucosal filler for ESD procedures.

## Materials and methods

### Reagents and animal

The novel submucosal filler developed by Shandong Weigao Pharmaceutical Co., Ltd. Owing to patent–related considerations, the novel submucosal filler contains ingredients acetylcysteine, hydroxyethyl starch, sodium chloride, water, and methylene blue, but the detailed concentration ratios are not fully disclosed. While nebulized acetylcysteine administered at concentrations of 10–20% is clinically viable, this drug is associated with gastrointestinal side effects such as nausea and vomiting, thus, to strike a balance between efficacy and safety, the 10% concentration was prioritized. Furthermore, our preliminary experiments confirmed that a formulation containing 10% acetylcysteine as the primary component is optimal for facilitating the submucosal dissection process, with the supporting data from these experiments documented in S1 Table. A total of eight healthy Guangxi Bama miniature pigs, weighing 30–35 kg, were used in this study, which was conducted from July 2021 to August 2021. All experiments were performed at the Laboratory Animal Center of Weihai Desheng Technology Testing Co., Ltd. This study was reported in accordance with ARRIVE guidelines. Prior to the experiment, this study was reviewed and approved in accordance with the guidelines of the Institutional Animal Care and Use Committee (IACUC) of Weihai Desheng Technology Testing Co., Ltd., on July 20, 2021 (Approval No. M2021072011).

### Study design

#### Negative control group.

**Fig 1:**
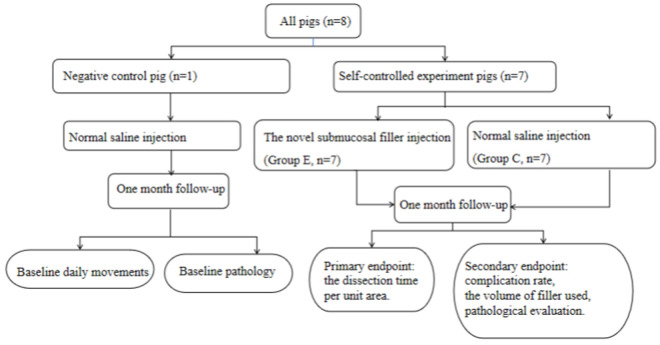
Flowchart of research procedure.

A single pig was randomly selected for ESD (Fig 1). The esophageal wall, at 40 cm or 45 cm from the incisors, was selected as the site. Only normal saline was used for the submucosal injection. The pig’s daily living conditions were monitored, and the pathological status of the esophageal wound healing, along with heart, liver, and kidneys was examined one month after ESD.

#### Self-controlled experiment group (Group E VS. Group C).

Seven pigs were randomly selected to undergo ESD (Fig 1). The ESD procedure was performed on the same side of the esophagus, at either 40 cm or 45 cm from the incisors. This selection was made to avoid potential variations in procedural difficulty associated with different ESD sites. By maintaining a certain distance between the two sets of treatment areas, it was easier to observe the wound locations and associated complications in Group E and Group C post-operation. Two highly experienced endoscopists, each having performed over 500 ESD cases, alternated in conducting the ESD procedures on the same pig, there was no obvious basis for them to compare differences in the difficulty of submucosal dissection on the same individual. This approach minimized the variability in decision-making between operators. One month after ESD, an evaluation was carried out to assess the long-term safety of the filler. The evaluation focused on aspects such as the healing of ESD -induced wounds and the function of the heart, liver, and kidneys.

### Procedure

#### Preoperative preparation.

Prior to the ESD procedure, the pigs were fasted for three days. During this fasting period, their nutritional requirements were met through the administration of a sugar-salt water solution in physiologically appropriate amounts.

#### Anesthesia method.

Twelve hours prior to the induction of anesthesia, the pigs were subjected to fasting. Thirty minutes before the commencement of anesthesia induction, the pigs were carefully weighed. Anesthesia was initiated via an intramuscular injection of 1.5 ml of Shutai 50. Subsequently, the pigs were exposed to 5% isoflurane inhalation. This process continued until satisfactory muscle relaxation and adequate sedation were achieved, at which point tracheal intubation was promptly performed. During the entire procedure, anesthesia was maintained using 1–2% isoflurane to ensure the pigs’ stable anesthetic state.

#### Instruments and descriptions of ESD (Group C: Video 1 VS. Group E: Video 2).

The endoscopists use a therapeutic endoscope (Olympus GIF-HQ290J, Japan) and disposable mucosal resection knife (Olympus KD-650Q, Japan), 23-gauge needle (Olympus NM-400V-0423, Japan), hot biopsy forceps (Olympus FD-410LR, Japan) and an electrical generator (ERBE 300D, Germany). The procedure followed standard ESD steps including marking, mucosal incision, submucosal dissection and hemostasis. All submucosal fillers are mixed with methylene blue, appearing as a light-blue liquid for better depiction of margins and submucosal tissue. For the ESD procedure, two types of submucosal fillers were used. The esophageal wall, located either 40 cm or 45 cm from the incisors on the same side of the same pig, was randomly selected as the ESD site. The pigs were then assigned into two groups: Group E, which received the novel submucosal filler and Group C, which received normal saline. Both endoscopists were kept blinded to the type of submucosal filler used.

#### Postoperative care and endoscopy follow-up.

After the ESD, the pigs were fasted on the first postoperative day. On the second day, they were provided with sugar-salt water. By the third day, a semiliquid diet was introduced. Oral lansoprazole rapidly disintegrating tablets and cefixime dispersible tablets were administered to the pigs for a three-day course. Throughout the postoperative period, detailed records were maintained, including the pigs’ diet intake, body temperature, bowel movement patterns, and activity levels. One month after the ESD procedure, an endoscopic examination was performed to assess the wound healing status. Upon completion of the endoscopic re-examination, the pigs were humanely euthanized. Subsequently, anatomical dissections were carried out. Tissue samples were carefully collected from relevant organs and the esophageal wound site.

#### Self-controlled experiment group blood tests.

Routine blood work along with serum biochemical tests were carried out one day before and one week after ESD.

#### Pathological assessment with Hematoxylin and Eosin (H&E) Methods.

All pathological sections were independently reviewed in a blinded manner by two senior pathologists. In cases of divergent opinions, a third expert pathologist conducted a re-evaluation in a blinded manner to resolve the discrepancies.

(i) The thickness of the submucosal layer in the specimens from Group E and Group C was measured.
(ii) Wound healing from Group E and Group C was classified according to the maturity of the regenerative mucosal tissue:
  Excellent: The epithelium was intact, with well-formed structures, abundant capillaries, and minimal infiltration of inflammatory cells, earning a grade 2.
  Good: The epithelial integrity was compromised, the structure was disorganized, fewer capillaries and moderate infiltration of inflammatory cells, resulting in a Grade 1.
  Poor: There were only a few nascent epithelial cells, leading to a Grade 0.
(iii) Pathological evaluations of heart, liver, and kidneys tissues were also conducted for comparison between the negative control group and the self-controlled experiment group.

### Outcome measurements

The primary focus of our analysis was the dissection time per unit area. The dissection time per unit area was calculated by dividing the dissection time by the area (min/cm^2^). The mucocal dissection time defined as the duration from mucocal marking until the lesion was completely detached form all tissue attachments. The dissection areas marked on the two parts were intended to be as identical as possible. However, in both the live porcine model experiments and clinical practice, the endoscopist’s procedures could not be maintained completely consistent. Thus, the specimen area was calculated by multiplying the longest and shortest diameters (cm^2^).

Secondary study endpoints encompassed the complication rate, the volume of submucosal filler per unit area (calculated by dividing the total filler volume by the area), pathological assessment of the submucosal layer thickness in specimens, histologic evaluation of wound healing, as well as examination of the heart, liver, and kidneys. Complications were categorized as follows: introperative perforation and active bleeding requiring titanium clip intervention, delayed postoperative perforation, delayed bleeding, decrease in hemoglobin of> 2g/dL or confirmed significant hemorrhage within 1 week after ESD; and stricture, defined as a condition where a 9–10 mm endoscope was unable to pass through the esophagus.

### Statistical analyses

Statistical analyses were performed using SPSS version 26.0, and graphs were created with OriginPro 2024 software. The Shapiro-Wilk test was used to assess the normality of data distribution. Study data are expressed as median and interquartile range (IQR, P25, P75) for skewed distribution continuous variables, mean ± standard deviation (SD) for normally distributed continuous variables, and frequencies and percentages for qualitative variables. The paired t-test was used for comparisons involving normally distributed continuous variables, and the Wilcoxon signed-rank test was applied for skewed distributed continuous variables. A P value < 0.05 was regarded as statistically significant.

## Results

### Negative control survival pig experiment

The pig was injected submucosally with normal saline during ESD.The pig was kept alive for one month and then sacrificed for dissection. The pathological examination of the heart, liver, kidneys, and wound area served as a negative control for histological evaluation. Throughout the post operative survival period, the pig’s diet, body temperature, bowel movements, and activity levels remained normal.

### Self-controlled experiment survival pig experiment

Seven pigs underwent a self-controlled experiment (Table 1). During the entire post operative period of care, their diet, body temperature, bowel movements, and activity levels remained normal, which was comparable to those of the negative control survival pig.

**Table 1:** ESD data and wound pathology of the pigs between Group E and Group C.

Pig number	Group	ESD site	Time (min)	Area (cm^2^)	Efficiency(min/cm^2^)	Thickness (mm)	The volume of filler (ml/cm^2^)	Wound pathology
1	C	45	20	3.24	6.17	0.01	15.43	Grade 0
1	E	40	16	3.61	4.43	0.1	3.88	Grade 0
2	E	45	16	2.79	5.73	0.18	3.94	Grade 2
2	C	40	12	1.62	7.41	0.27	8.02	Grade 2
3	E	45	8	2.66	3.01	0.1	5.45	Grade 2
3	C	40	9	1.97	4.57	0.25	4.57	Grade 2
4	E	45	10	2.62	3.82	0.14	2.29	Grade 2
4	C	40	7	2.87	2.44	0.14	3.66	Grade 2
5	C	45	13	1.96	6.63	0.1	4.85	Grade 2
5	E	40	10	4.21	2.38	0.15	3.33	Grade 2
6	C	45	11	1.85	5.95	0.05	7.57	Grade 1
6	E	40	14	2.62	5.34	0.13	3.05	Grade 2
7	C	45	9	1.51	5.96	0.11	8.28	Grade 2
7	E	40	9	2.83	3.18	0.45	2.83	Grade 2

Note. ESD site is located in the esophageal at distances of 40 cm or 45 cm from the incisors.

Time is the dissection time (min: minute).

Area is the dissection area(cm^2^).

Efficiency is the dissection time per unit area(min/cm^2^).

Thickness is the submucosal thickness of the specimen(mm).

The pathological grade was esophagus ESD wound healing grade.

**Fig 2:**
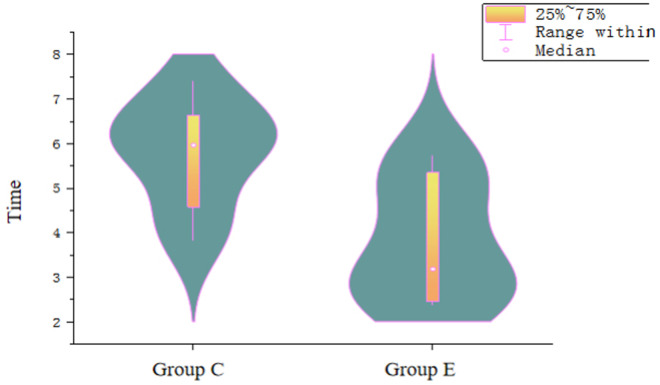
The dissection time per unit area was significantly shorter in group E than group C (minute).

In group E compared to Group C, the dissection time per unit area was significantly shorter (3.79 ± 1.38 minutes vs. 5.79 ± 1.22 minutes; *P* = 0.004). (Fig 2, Table 2), the dissection area was larger (2.79 cm^2^ vs. 1.96 cm^2^; *P* = 0.028), and the volume of filler used per unit area was significantly less (3.48 ± 1.07 ml vs. 7.48 ± 3.96 ml; *P* = 0.039). However, there were no significant differences in entire dissection time (11.86 ± 3.39 minutes vs. 11.57 ± 4.24minutes; *P* = 0.818), the thickness of the submucosal layer in the specimens (0.13 ± 0.03 mm vs. 0.18 ± 0.15 mm; *P* = 0.415).

**Table 2:** Comparison of experimental data between Group E and Group C.

	Group E (n = 7)	Group C (n = 7)	T/Z value	*P* value
Dissection area (cm^2^)	2.79 (2.62,3.61)	1.96 (1.62,2.87)	2.197	0.028
Dissection time (min)	11.86 ± 3.39	11.57 ± 4.24	−0.24	0.818
The dissection time per unit area (min)	3.79 ± 1.38	5.79 ± 1.22	4.485	0.004
The amount of filler used per unit area (ml)	3.48 ± 1.07	7.48 ± 3.96	−2.637	0.039
The submucosal thickness (mm)	0.13 ± 0.03	0.18 ± 0.15	0.876	0.415
Wound healing grade				
0	1(14.29%)	1(14.29%)		
1	0	1(14.29%)	−1.000	0.317
2	6(85.71%)	5(71.42%)		

### Complications

**Fig 3:**
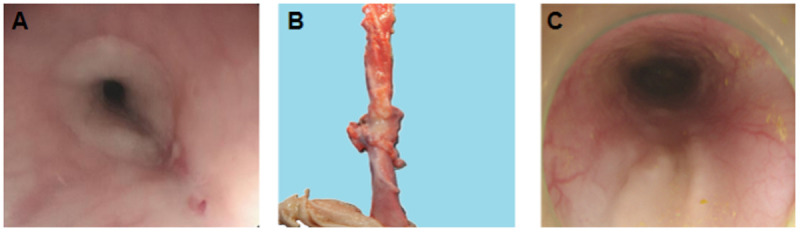
A. The endoscopic image show the stricture of pig’s (the number of one) ESD site. **B.** The anatomical image show the dissected pig’s (the number of one) two sites esophagus ESD wounds appeared rigid. **C.** The endoscopic image show other pigs’ well-healed wounds.

In both group E and Group C, there were no cases of perforation, and all the cases experienced slight bleeding but there was no requirement for titanium clip intervention. In one out of seven pigs, severe esophageal stricture occurred such that the endoscope could not pass through, subsequent anatomical examination revealed that the esophageal ESD wounds in both Group E and Group C showed similar stenosis and rigidity (Fig 3A and 3B), other pigs’ well-healed wounds (Fig 3C). The complication rates were comparable between Group E and Group C (Table 2).

### Self-controlled blood tests

Routine blood tests and serum biochemical tests were performed one day before and one week after ESD to enable a preoperative-postoperative comparison for the self-controlled experiment survival pigs. In this group, there was no significant increase in white blood cell ((22.21 ± 4.56)×10^9^/L vs. (24.62 ± 5.20)×10^9^/L; *P* = 0.312). However, the neutrophil count ((2.94 ± 1.28)×10^9^/L vs. (9.10 ± 5.73)×10^9^/L; *P* = 0.044), neutrophil percentage (12.94 ± 3.71% vs. 34.89 ± 15.54%; *P* = 0.02), platelet count ((450.00 ± 77.88)×10^9^/L vs. (530.57 ± 96.96)×10^9^/L; *P* = 0.024), monocyte count ((1.05 ± 0.20)×10^9^/L vs. (1.59 ± 0.31)×10^9^/L; *P* = 0.006), and monocyte percentage (4.77 ± 0.60% vs. 6.70 ± 1.90%; *P* = 0.002) all showed significant increases. Conversely, in the same group, the red blood cell count ((9.44 ± 0.81)×10^12^/L vs. (8.49 ± 0.56)×10^12^/L; *P* = 0.018), hemoglobin (161.71 ± 14.51 g/L vs. 145.29 ± 7.02 g/L; *P* = 0.009), hematocrit (51.50 ± 4.49% vs. 46.13 ± 2.39%; *P* = 0.013), lymphocyte count ((17.22 ± 3.21)×10^9^/L vs. (13.25 ± 2.56)×10^9^/L; *P* = 0.016), and lymphocyte percentage (77.79 ± 3.61% vs. 55.51 ± 13.58%; *P* = 0.011) all demonstrated significant decreases between one day before and one week after ESD.

In the same group, only albumin (16.91 ± 1.17 g/L vs. 14.39 ± 1.16 g/L; *P* = 0.045) showed significant decreases between one day before and one week after ESD. Aspartate aminotransferase (38.00 ± 14.01U/L vs. 29.14 ± 11.07U/L; *P* = 0.623), alanine aminotransferase (66.43 ± 6.95U/L vs. 49.43 ± 6.37U/L; *P* = 0.953), gamma-glutamyl transferase (57.14 ± 2.12U/L vs. 46.71 ± 4.99U/L; *P* = 0.966), blood urea nitrogen (3.16 ± 0.67 mmol/L vs. 3.36 ± 1.08 mmol/L; *P* = 0.497), total cholesterol (3.07 ± 0.50 mmol/L vs. 2.59 ± 0.49 mmol/L; *P* = 0.120), creatinine (103.59 ± 14.43umol/L vs. 83.31 ± 11.38umol/L; *P* = 0.200), alkaline phosphatase (98.00 ± 55.83U/L vs. 72.29 ± 24.64U/L; *P* = 0.325), blood glucose (4.77 ± 1.55U/L vs. 4.43 ± 1.09U/L; *P* = 0.559), total protein (83.76 ± 4.49g/L vs. 79.29 ± 5.93g/L; *P* = 0.208), and triglyceride (0.50umol/L vs. 0.041umol/L; *P* = 0.866) all showed no significant decreases between one day before and one week after ESD.

### Pathological evaluation

#### Wound’s pathological evaluation.

**Fig 4:**
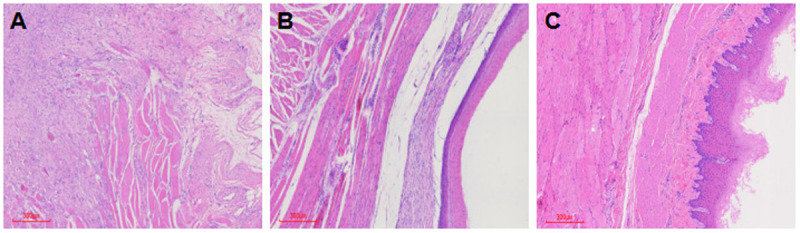
A. The pathological assessment shows the stricture of one pig’s Group E and Group C wound healing which showing grade 0. **B.** The pathological assessment shows one pig’s Group C wound healing which showing grade 1. **C.** The pathological assessment shows other pigs’ Group E and Group C wounds healing which showing grade 2. (The mucosal side is on the right and the outer side is on the left).

In one pig, the esophageal wounds in both Group E and Group C jointly presented a severe esophageal stricture. Pathological evaluation of the wound revealed a grade 0 (Fig 4A), which was in line with reparative changes subsequent to perforation. Histological findings demonstrated partial loss of esophageal mucosal epithelium, discontinuity of the muscularis propria, and extensive fibrosis with some inflammatory and multinucleated giant cell infiltration. In another pig from Group C, the wound pathological evaluation showed a grade 1 (Fig 4B), while the other esophageal wounds healed at grade 2 (Fig 4C). There were no significant differences in the epithelial structure and the degree of inflammatory cell infiltration of wound healing grade between Group E and Group C, the grade 2 wound – healing rate (85.71% vs. 71.42%; *P* = 0.317) (Table 2).

#### Cardiac, hepatic, and renal pathology.

There were no significant differences in the pathological evaluation between the negative control group, which was administered only normal saline, and the self-controlled group, in which all pigs received the novel submucosal filler. In the cardiac tissue, no pathological alterations were observed. Liver pathology indicated minimal to mild focal lymphocytic infiltration in portal area, along with minimal to mild hepatic sinusoidal congestion. Renal pathology revealed minimal to mild infiltration of interstitial inflammatory cell.

## Discussion

The current results clearly demonstrate the efficacy of the novel submucocal filler in an in vivo porcine model of esophagus ESD. To date, this particular substance (acetylcysteine) has not been reported as a submucosal filler for ESD. This study reveals that the novel filler can accelerate the esophageal ESD process without increasing the risk of complications.

The esophageal tissue is thinner compared to the gastric and colorectal tissues. Esophageal ESD is affected by the restricted working space, heartbeat and respiratory movements. These factors render esophageal ESD a more challenging procedure. This novel filler significantly expedite submucosal dissection (shown in Table 2) by utilizing the blunt mechanical separation effect generated through disrupting disulfide bonds, at the timestamp of 01:42 in Video 2 of the experimental group, the most authentic situation of spontaneous submucosal dissection during the movement of the electrosurgical knife can be observed. These videos could gain a clearer and more comprehensive understanding of the objective results of the experiment and the visible effects presented. This advantage can also be applied to ESD-related technologies, such as peroral endoscopic myotomy and endoscopic submucosal tunnel dissection.

Blunt dissection has two main advantages. Firstly, it offers clearer visualization and easier management of blood vessels, which reduces the risk of intraoperative perforation and bleeding. Secondly, it minimizes thermal damage from electrosurgery to the muscularis propria, thereby decreasing the risk of delayed perforation, bleeding, electrocoagulation syndrome [5], and severe stenosis [9]. This study reveals clinically important findings, showing that the Group E required shorter dissection time compared to Group C (3.79 ± 1.38 minutes vs. 5.79 ± 1.22 minutes; *P* = 0.004). This improvement in efficiency also eliminated the need for repeated submucosal filler injection and the volume of filler used per unit area was reduced (3.48 ± 1.07 ml vs. 7.48 ± 3.96 ml; *P* = 0.039). Importantly, the complication rates in Group E and Group C were found to be equal. The disruption of disulfide bonds occurs at the molecular level and has a negligible histological impact [10]. This is evidenced by the similar histological evaluations of the specimens from Group E and Group C. Additionally, the similar submucosal layer thickness in Group E and Group C (0.13 ± 0.03 mm vs. 0.18 ± 0.15 mm; *P* = 0.415) validates that the filler does not cause the loss of submucosal layer, ensuring the accuracy of vertical margin histological evaluation remains unaffected. The vertical margin pathology of the submucosal layer in ESD specimens is analyzed to determine whether the resection is complete and if further surgery is required. Consequently, the preservation of the submucosal layer is crucial for vertical margin pathological evaluation.

One month post-ESD, both endoscopic and pathological assessments of wound healing showed similar grade 2 healing rates (85.71% vs. 71.42%; *P* = 0.317) between the Group E and Group C. There are abundant arterioles, venules, and lymphatic vessels in the submucosa. When this filler is used for submucosal injection, there is still the issue of absorption through the blood and lymphatic circulation. It is of certain significance to evaluate the functions and pathological evaluations of important organs to rule out systemic toxicity. Pre and post operative routine blood tests and serum biochemical tests mainly indicated nutritional deficiencies, as well as inflammatory response and stress state induced by ESD. Moreover, there were no significant differences in cardiac, hepatic, and renal pathology between the negative control group and the self-controlled group. Overall, the above results suggest that the novel filler is safe, with no adverse effects on wound healing or vital organ function.

Numerous studies have explored submucosal injection materials from diverse perspectives. In 2008, the Sumiyama team reported on the chemical peeling effects of mesna submucosal injections in gastric and esophageal ESD [11–14]. However, in an animal peroral endoscopic myotomy experiment, the incidence of postoperative abscess was higher in the mesna- treated group [13]. ORISE gel has been linked to extensive foreign body reactions and granuloma formation with fibrosis and unabsorbed gels present a distinct amyloid or mucoid appearance, which can affect pathological diagnostics [15,16]. Boston Scientifific voluntarily issued a FDA Class II device recall of ORISE gel [17]. In contrast, in our present study, neither abscess formation nor foreign body reactions were detected during the anatomical or pathological evaluation of the tissue. Another submucosal injection material, SIC-8000, has been limited by reported bubbling during submucosal dissection, which can interfere with the visualization [17]. Notably, no such issue was observed in our investigation. This suggests that the filler employed in our study may offer certain advantages over these previously studied submucosal injection materials, potentially providing a more favorable option for endoscopic procedures in terms of safety and visualization.

Other researches have explored the utilization of viscous solutions, aiming to achieve lower lateral fluid diffusion and prolong the duration of the submucosal cushion. These characteristics allow a reduction in procedural time to complete the procedure. Nowadays, several novel submucosal fillers are under investigation, including the Na-CMS gel [2], G-OALG hydrogel [18], PBA-ApGltn [19], Gellan gum [20], Chitosan hydrogel [21], and Sodium alginate [22–24]. However, the poor long-term stability and the complexity of temporary preparation of some hydrogels pose additional challenges for their mass production and application [25]. Synthetic polymers often carry the risk of triggering unintended and adverse biological reactions [26]. One of the major concerns regarding the routine use of viscous solutions lies in their cost [27]. For instance, substances like sodium hyaluronate [2,24] and fibrin glue [28] are costly, and a special syringe has even been developed to extrude these high viscosity solutions [29]. Moreover, hydrogels may prematurely clog inside the long tube, making smooth injection more challenging [26]. In addition, submucosal elevation is created at an unintended site or if there is excessive injection, the submucosal cushion becomes durable and irreducible, which might continue to interfere with the procedure, especially in areas with narrow operation fields like the esophagogastric junction and pyloric ring [30]. Another study introduced a two-step injection system comprising 2.0% calcium chloride solution and 0.4% sodium alginate solution, achieved high performance in ex vivo models [30]. Nevertheless, further evaluations are warranted to determine whether the two-step injection fluids can be administered through an ESD knife with injection capability [29]. The novel submucosal filler explored in our study offers unique advantages. It is user-friendly, cost-effective, readily accessible, and exhibits excellent syringeability. These findings strongly support the potential of this novel submucosal filler for gastrointestinal ESD in real-world clinical settings, and the implications warrant further clinical studies. We anticipate that this filler will represent a meaningful advancement, making ESD and ESD-derived techniques easier to perform and more widely applicable. This could be particularly beneficial for complex ESD cases, such as those involving the duodenum, scar tissue, or lesions with severe infiltration, where it can offer great promise.

Our study does have several limitations. Firstly, the biological as well as molecular mechanism of action of this novel filler have not yet been investigated, which constitutes a limitation of the present study, and the sample size was insufficient. Although the effect was remarkable enough to obviate the necessity of repeating the ESD operation with a large sample size, these limitations might have potentially led to an underestimation of the complication incidence. As a self-controlled experiment, it inherently bears certain limitations that were beyond our capacity to alter during this research. In future investigations, well-designed, fully randomized experiments should be considered, and it is crucial that we remain committed to advancing step by step toward deeper mechanistic research.

Secondly, in our current study, pigs only underwent a single endoscopic re-examination one month after ESD. This approach left our understanding of wound healing at each stage insufficient. To address this, larger sample research with more precise experimental designs, and longer follow-up periods are needed. For example, endoscopy was performed weekly over a four week period to monitor the processes of wound healing and any delayed complications. We will also conduct biological experiments that will help us gain a comprehensive understanding of the effects and impacts of this novel filler on esophageal cells. Specifically, these experiments will allow us to investigate whether the filler promotes epithelial migration and fibroblast-mediated repair, thereby providing mechanistic evidence for its role in wound healing following endoscopic submucosal dissection. This will be a key focus of our future research, particularly as this line of inquiry is closely linked to esophageal wound stricture—a critical clinical issue that demands attention.

Thirdly, the scope of this study was confined to esophageal ESD. Given the anatomical and tissue-level disparities across the gastrointestinal tract, it is possible that the novel submucosal filler’s performance characteristics could vary in other regions.

Fourthly, due to patent–related considerations, the detailed concentration ratios of the novel submucosal filler have not been fully disclosed, It should be emphasized that the study focuses on evaluating the safety and efficacy of the filler rather than exact replication. Despite using a live porcine model in this experimental study, which closely mimics clinical practice in terms of having blood flow, peristalsis, and respiratory movement, and with digestive structures that are histologically and functionally similar to humans [22,31], and long-term survival tests were also conducted, further studies involving human patient is essential.

## Conclusion

The novel submucosal filler has proven to be both effective and safe in the porcine model. It significantly alleviates the challenges associated with submucosal dissection, thereby expediting the ESD process without elevating the risk of complications.

## Supporting information

S1 Table Selection of optimal concentration for the filler (preliminary experiment data). (DOCX)

## References

[1] Aihara H, Othman MO, Jawaid SA, Gorgun E, Sharma NR, Siddiqui UD, et al. A multicenter, retrospective study of a through-the-needle injection-capable electrosurgical knife for endoscopic submucosal dissection. Gastrointest Endosc. 2024;100(6):1034–42. doi: 10.1016/j.gie.2024.06.011 38879045

[2] Chen C, Liu XY, Cheng CE, Xiong YJ, Sun YB, Tan CH, et al. Efficacy and safety of a novel submucosal injection solution for endoscopic resection in porcine models. J Dig Dis. 2021;22(1):49–56. doi: 10.1111/1751-2980.12963 33236832

[3] Liu Y, Li R, Tan C, Ma Y, Feng J, Xu Q, et al. Application of Hemocoagulase Bothrops Atrox in the submucosal injection for endoscopic submucosal dissection: a preliminary trial. Eur J Gastroenterol Hepatol. 2021;33(1S Suppl 1):e681–5. doi: 10.1097/MEG.0000000000002206 34034279 PMC8734620

[4] Furube T, Takeuchi M, Kawakubo H, Maeda Y, Matsuda S, Fukuda K, et al. The relationship between the esophageal endoscopic submucosal dissection technical difficulty and its intraoperative process. Esophagus. 2023;20(2):264–71. doi: 10.1007/s10388-022-00974-x 36508068

[5] Qiu J, Ouyang Q, Zhang Y, Xu J, Xie Y, Wei W, et al. Post-endoscopic submucosal dissection electrocoagulation syndrome: a clinical overview. Expert Rev Gastroenterol Hepatol. 2022;16(11–12):1079–87. doi: 10.1080/17474124.2022.2156858 36503328

[6] Koyama Y, Fukuzawa M, Aikawa H, Nemoto D, Muramatsu T, Matsumoto T, et al. Underwater endoscopic submucosal dissection for colorectal tumors decreases the incidence of post-electrocoagulation syndrome. J Gastroenterol Hepatol. 2023;38(9):1566–75. doi: 10.1111/jgh.16259 37321649

[7] Raghu G, Berk M, Campochiaro PA, Jaeschke H, Marenzi G, Richeldi L, et al. The multifaceted therapeutic role of N-acetylcysteine (NAC) in disorders characterized by oxidative stress. Curr Neuropharmacol. 2021;19(8):1202–24. doi: 10.2174/1570159X19666201230144109 33380301 PMC8719286

[8] Rhee CK, Lim SY, Lee W-Y, Jung JY, Park YB, Lee CY, et al. The effect of nebulized N-acetylcysteine on the phlegm of chronic obstructive pulmonary disease: the NEWEST study. BMC Pulm Med. 2024;24(1):434. doi: 10.1186/s12890-024-03243-y 39223526 PMC11369990

[9] Zhou X, Chen H, Chen M, Ding C, Zhang G, Si X. Comparison of endoscopic injection of botulinum toxin and steroids immediately after endoscopic submucosal dissection to prevent esophageal stricture: a prospective cohort study. J Cancer. 2021;12(19):5789–96. doi: 10.7150/jca.60720 34475992 PMC8408129

[10] Sumiyama K, Tajiri H, Gostout CJ, Kawamura M, Imazu H, Ohya TR, et al. Chemically assisted submucosal injection facilitates endoscopic submucosal dissection of gastric neoplasms. Endoscopy. 2010;42(8):627–32. doi: 10.1055/s-0029-1244223 20552541

[11] Dobashi A, Goda K, Sumiyama K, Kobayashi M, Ohya TR, Kato M, et al. A feasibility study of chemically assisted endoscopic submucosal mechanical dissection using mesna for superficial esophageal squamous cell carcinomas. Surg Endosc. 2015;29(11):3373–81. doi: 10.1007/s00464-014-4031-7 25515984

[12] Sumiyama K, Gostout CJ, Rajan E, Bakken TA, Knipschield MA. Chemically assisted endoscopic mechanical submucosal dissection by using mesna. Gastrointest Endosc. 2008;67(3):534–8. doi: 10.1016/j.gie.2007.10.041 18294517

[13] Kawahara Y, Sumiyama K, Tajiri H. Chemically assisted peroral endoscopic myotomy with submucosal mesna injection in a porcine model. Minim Invasive Ther Allied Technol. 2015;24(6):334–9. doi: 10.3109/13645706.2015.1040419 25921483

[14] Sumiyama K, Toyoizumi H, Ohya TR, Dobashi A, Hino S, Kobayashi M, et al. A double-blind, block-randomized, placebo-controlled trial to identify the chemical assistance effect of mesna submucosal injection for gastric endoscopic submucosal dissection. Gastrointest Endosc. 2014;79(5):756–64. doi: 10.1016/j.gie.2013.09.027 24238308

[15] Sun BL. Submucosal lifting agent ORISE gel causes extensive foreign body granuloma post endoscopic resection. Int J Colorectal Dis. 2021;36(2):419–22. doi: 10.1007/s00384-020-03764-y 32959116

[16] Ibarra-Arzamendia PN, Hanly MG. Histopathological findings related to ORISE™ injectable submucosa lifting agent used in the endoscopic mucosal resection of bowel neoplasms: a review of three cases. Case Rep Pathol. 2020;2020:6918093. doi: 10.1155/2020/6918093 32082675 PMC7013285

[17] ASGE Technology Committee, Bhatt A, Bucobo JC, Abdi M, Akshintala VS, Chen D, et al. Submucosal injection fluid and tattoo agents. Gastrointest Endosc. 2024;100(5):797–806. doi: 10.1016/j.gie.2024.07.002 39269377

[18] Fan C, Xu K, Huang Y, Liu S, Wang T, Wang W, et al. Viscosity and degradation controlled injectable hydrogel for esophageal endoscopic submucosal dissection. Bioact Mater. 2020;6(4):1150–62. doi: 10.1016/j.bioactmat.2020.09.028 33134608 PMC7588753

[19] Nagasaka K, Komatsu H, Ito S, Palai D, Nishiguchi A, Taguchi T. Elevation and adhesion properties of injectable hydrogels based on catechol/boronic acid-modified Alaska pollock gelatin for endoscopic submucosal dissection. Colloids Surf B Biointerfaces. 2025;245:114307. doi: 10.1016/j.colsurfb.2024.114307 39405947

[20] Tang Y, Hu M, Tang F, Huang R, Wang H, Wu D, et al. Easily-injectable shear-thinning hydrogel provides long-lasting submucosal barrier for gastrointestinal endoscopic surgery. Bioact Mater. 2021;15:44–52. doi: 10.1016/j.bioactmat.2021.11.026 35386335 PMC8940951

[21] Jeon HJ, Choi HS, Bang EJ, Lee KW, Kim SH, Lee JM, et al. Efficacy and safety of a thermosensitive hydrogel for endoscopic submucosal dissection: an in vivo swine study. PLoS One. 2021;16(12):e0260458. doi: 10.1371/journal.pone.0260458 34882721 PMC8659419

[22] Jung KU, Lee YJ, Jang J-Y, Cho JY. Efficacy and safety of a submucosal injection solution of sodium alginate for endoscopic resection in a porcine model. Sci Rep. 2024;14(1):4592. doi: 10.1038/s41598-024-55226-y 38409310 PMC10897473

[23] Lee AY, Jang JY, Seo J-Y, Kim SH, Choi JM, Cho JY. Efficacy and safety of MC-003 solution for endoscopic mucosal or submucosal resection: a prospective, multicenter, randomized, triple-blinded, parallel-group, phase III study. Gastrointest Endosc. 2024;100(1):36-45.e1. doi: 10.1016/j.gie.2024.01.002 38184114

[24] Kawaguchi K, Isomoto H. Novel submucosal injection solution 0.6% sodium alginate: Bench to market for endoscopic submucosal dissection optimization. Dig Endosc. 2019;31(4):393–5. doi: 10.1111/den.13426 31034664

[25] Ni P, Ye S, Li R, Shan J, Yuan T, Liang J, et al. Chitosan thermosensitive hydrogels based on lyophilizate powders demonstrate significant potential for clinical use in endoscopic submucosal dissection procedures. Int J Biol Macromol. 2021;184:593–603. doi: 10.1016/j.ijbiomac.2021.06.111 34174301

[26] Chen Z, Ding J, Wu C, Wei D, Sun J, Fan H, et al. A review of hydrogels used in endoscopic submucosal dissection for intraoperative submucosal cushions and postoperative management. Regen Biomater. 2023;10:rbad064. doi: 10.1093/rb/rbad064 37501677 PMC10368804

[27] Lisotti A, Marocchi G, Calì A, Fusaroli P. Endoscopic mucosal resection of large colonic laterally spreading tumors using a dedicated viscous solution for submucosal injection (ORISE gel): a short case series (with video). Eur J Gastroenterol Hepatol. 2021;33(5):650–4. doi: 10.1097/MEG.0000000000002014 33323756

[28] Takao M, Takegawa Y, Takao T, Sakaguchi H, Nakano Y, Tanaka S, et al. Fibrin glue: novel submucosal injection agent for endoscopic submucosal dissection. Endosc Int Open. 2021;9(3):E319–23. doi: 10.1055/a-1315-0059 33655028 PMC7892272

[29] Abe S, Bhatt A, Saito Y. The journey to develop the ideal submucosal injection solution for endoscopic submucosal dissection. Gastrointest Endosc. 2021;93(2):514–6. doi: 10.1016/j.gie.2020.07.027 33478668

[30] Hirose R, Nakaya T, Naito Y, Yoshida T, Bandou R, Daidoji T, et al. An innovative next-generation endoscopic submucosal injection material with a 2-step injection system (with video). Gastrointest Endosc. 2021;93(2):503-513.e5. doi: 10.1016/j.gie.2020.06.031 32565186

[31] Giannino V, Salandin L, Macelloni C, Longo LM. Evaluation of Eleview(®) bioadhesive properties and cushion-forming ability. Polymers (Basel). 2020;12(2). 10.3390/polym12020346 PMC7077458 32033452

