# Correction: An innovative submucosal filler for esophageal endoscopic submucosal dissection: A porcine model study (with video)

**DOI:** 10.1371/journal.pone.0339320

**Published:** 2025-12-18

**Authors:** LiLin Lin, SuYu Chen, Rui Huang, JianHua Chen, Juan Lin, Hong Shi

In the Competing interest’s statement, the patent application number is listed incorrectly. The correct patent application number is: 202411330048.9.
