# Supplementary material for: An innovative submucosal filler for esophageal endoscopic submucosal dissection: A porcine model study (with video)
Source: PLoS One. 2025 Sep 11;20(9):e0331618. doi: 10.1371/journal.pone.0331618 (PMC12425303; doi:10.1371/journal.pone.0331618)
Supplement: S1 Table — (DOCX) [file pone.0331618.s001.docx]

**S1 Table Selection of Optimal Concentration for the Filler (Preliminary Experiment Data)**

| **Pig**  **number** | **ESD**  **site** | **Filler Concentration** | **Time (min)** | **Area**  **(cm^2^)** | **Efficiency(min/cm^2^)** | **Thickness(mm)** |
| --- | --- | --- | --- | --- | --- | --- |
| A | 40 | Normal saline | 17 | 2.66 | 6.40 | 0.05 |
| B | 50 | 3% acetylcysteine | 15 | 3.1 | 4.84 | 0.01 |
|  | 48 | 10% acetylcysteine | 13 | 4.23 | 3.07 | 0.18 |
|  | 45 | 8% acetylcysteine | 12 | 2.23 | 5.38 | 0.05 |
|  | 40 | 5% acetylcysteine | 12 | 2.34 | 5.13 | 0.3 |
|  | 35 | 7% acetylcysteine | 12 | 3.75 | 3.20 | 1 |

Note. The ESD site is located in the esophagus at a distance from the incisors.

Time is the dissection time (min: minute).

Area is the dissection area(cm^2^).

Efficiency is the dissection time per unit area(min/cm^2^).

Thickness is the submucosal thickness of the specimen(mm).
